# Ukrainian refugee crisis management in the Local Health Authority Roma 1: the challenges of implementing public health policies and lessons learned

**DOI:** 10.1186/s12889-023-15556-4

**Published:** 2023-07-10

**Authors:** Paolo Parente, Andriy Melnyk, Lavinia Camilla Barone, Maryana Kohut, Rosaria Messina, Paolo Lombardo, Leonardo Villani, Maria Teresa Riccardi, Maria Grazia Martelli, Adriano Grossi, Andrea Barbara, Valerio Mogini, Giancarlo Santone, Mauro Goletti

**Affiliations:** 1 Local Health Authority Roma 1, Borgo Santo Spirito, 3, Rome, 00193 Italy; 2 Section of Hygiene, University Department of Life Sciences and Public Health, Università Cattolica del Sacro Cuore, Largo Francesco Vito 1, Rome, 00168 Italy; 3 Department of Public Health and Infectious Diseases, Sapienza University of Rome, Piazzale Aldo Moro 5, Roma, 00185 Italy; 4 Division of Public Health, Italian Red Cross International, Roma, Italy

**Keywords:** Refugee, Emergency, Resources, Implementation, Resilience, Local Health Authority, Leadership, Team building, Multiculturalism

## Abstract

**Background:**

The conflict between Russia and Ukraine has strained the health systems of countries that welcome war refugees on all levels, from national to local. Despite the Public Health guidelines regarding assistance being published on the topic, the scientific literature currently lacks evidence on the experience of applying theory in practice. This study aims to describe evidence-based practices that were implemented and to provide a detailed description of emerging problems and solutions pertaining Ukrainian refugee assistance in the context of one of the biggest Local Health Authorities in Italy (LHA Roma 1).

**Methods:**

LHA Roma 1 developed a strategic plan based on local expertise, national and international guidelines to ensure infectious disease prevention and control, as well as continuity of care for non-communicable diseases and mental health.

**Results:**

The insertion of Ukrainian refugees in the National Health System through an identification code assignment and other services such as COVID-19 swab and vaccination were provided either in one of the three major assistance hubs or in local district level ambulatories spread throughout the LHA. Many challenges were faced during the implementation phase of the outlined practice guidelines, which required sensible and timely solutions. These challenges include the necessity of rapid resource provision, overcoming linguistic and cultural barriers, guaranteeing a standard of care across multiple sites and coordination of interventions. Public Private Partnerships, the creation of a centralized multicultural and multidisciplinary team and the mutually beneficial collaboration with the local Ukrainian community were essential to guarantee the success of all operations.

**Conclusions:**

The experience of LHA Roma 1 helps shed light on the importance of leadership in emergency settings and how a dynamic relationship between policy and practice would allow each intervention to be modulated according to the local environment, to better realize the potential of local realities to provide appropriate health interventions to all those in need.

## Background

Ensuring refugee access to healthcare services and their integration into local and national safety nets that provide safe housing, access to food and other fundamental necessities is an essential part of Public Health practice. Ukrainian refugees fleeing the current war face many obstacles. The refugee population represents a Public Health challenge given its different epidemiological and social backgrounds. Ukraine’s Noncommunicable Diseases (NCDs) burden [1], which is reflected, for example, in its high death rate for ischemic heart disease, diabetes prevalence, mental illness and alcohol-related deaths, will be further exacerbated by the current war, as refugees will face increased risk of complications for common NCDs due to a potential disruption in the supply of medications and timely access to specialist care. Indeed, in the last years Ukraine has been facing a humanitarian crisis affecting 5 million people, of whom 3.8 million are in need of emergency health services [2]. Moreover, major differences between Ukraine and Italy are evident when considering several indicators, such as routine vaccination coverage and prevalence of infectious diseases. For example, Ukraine reports suboptimal childhood vaccination rates for many indicators [3], such as DPT (78%) and HepB3 (77%), which are significantly lower than Italy (94% for both vaccinations). Similarly, Ukraine has the 4th highest tuberculosis incidence rate among the World Health Organization (WHO) European Region and the second highest prevalence of HIV/TB coinfection (26%) [4], with incidence rates significantly higher than Italy (71 per 100.000 people vs. 5 per 100.000) or HIV (0.3 vs. 0.04 per 1000 adults aged 15-49) [5].

In this context, several challenges faced by the refugees were addressed by the Italian government with specific measures aimed at providing temporary protection status, financial aid, transportation, free aid for identification and passports, eased access to jobs for certain professional profiles (i.e., doctors and nurses), access to education, and access to Italian healthcare service, which is provided by the Local Health Authorities (LHA) in the territory [6]. Furthermore, the current literature lacks evidence on local Public Health practice implementation of the guidelines developed to assist the refugees. The aim of this research in practice article is therefore to provide a detailed description of emerging problems and solutions pertaining to Ukrainian refugees assistance in the context of the Local Health Authority Roma 1, one of the biggest LHAs in Italy, and to describe evidence-based practices that were implemented to increase the resilience of our local health system to the current crisis in order to make available a replicable model in similar emergency situations.

## Main text

Starting on the 24th of February 2022, Ukraine has been invaded by Russian armed forces [7], so many Ukrainian citizens were forced to flee from their homes as refugees. In the first 4 and a half months, more than 139,000 Ukrainians arrived on Italian territory, the majority being women and children. Based on national data from the Italian Ministry of Internal Affairs, the distribution of temporary assistance requests is not evenly distributed throughout Italy, with a higher concentration in bigger cities, with Rome being the first by absolute number [8]. Furthermore, national data severely underestimates actual refugee flow, because not all the refugees request assistance through official channels. Most Regions in Italy are composed of multiple Local Health Authorities (LHAs), the functional units of public healthcare provision, which are responsible for guaranteeing the essential levels of care and health promotion for both residents and non-residents, free of charge. In particular, the Local Health Authority “ASL Roma 1” is one of the biggest in Italy, both in terms of population and geographical extension [9], and it contains critical urban nodes such as the Central Railway Station of Rome (“Termini” station) and the Minor Basil of Saint Sophie, one of the main religious and cultural centers for the Ukrainian Christian community. In such a context, particular attention needs to be given to people with high social vulnerability, such as unaccompanied minors, pregnant women, and single-parent families. Furthermore, major differences in the epidemiological status of the Ukrainian population, both in communicable and non-communicable diseases, compared to the national population represent a major challenge for a smooth integration process: for example, Ukraine is one of the countries with the lowest COVID-19 vaccination coverage (as of July 2022, 34% of the population compared to Italy’s 91%) [10, 11], which was a major focus of the LHA’s response.

### Initial planning and cooperation

To rapidly respond to the refugees’ needs, the LHA Roma 1 created a strategic plan to ensure infectious diseases prevention and control, as well as continuity of care for noncommunicable diseases and mental health issues for all who were assisted. The strategic plan was first developed based on local expertise and the necessity of immediate action, and later refined based on national and regional guidelines (12), the available literature, the European Centre for Disease Prevention and Control (ECDC) recommendations concerning Ukrainian refugees [13] and the feedback from the health professionals on the field. The services covered by this plan included first reception, COVID-19 antigenic testing and Tuberculosis screening through an anamnestic questionnaire; attribution of the Temporarily Present Foreigner (STP – Straniero Temporaneamente Presente) code and enrolment in the Italian National Health Service (NHS); evaluation of COVID-19 vaccination status; health and social needs assessment, orientation through an interview with a health professional; mental health support and social support to those who need it; activation of a tailored care pathway for people in critical health conditions or with specific health needs (oncological, diabetes treatment, needing life-saving medication, etc.). LHA Roma 1 has directly assisted more than 9300 refugees, issuing as many STP codes, and promptly providing socio-sanitary assistance to both parents and children who transit on its territory by setting up first reception centers to match everyone’s primary needs.

The release of an STP code represents, *de facto*, the moment of access to the Italian National Health Service for refugees, as it guarantees clinical treatment, urgent care, continuity of care for chronic disease and access to preventive medical procedures (e.g., maternity care, vaccinations, rehabilitation services) for those who lack a regular visa permit, on par with Italian citizens [14].

### Care pathways

The first point of contact for Ukrainian refugees with the National Health Service occurred at reception centers set up to respond to the emergency, either through a Central Reception Pathway (CRP) or a District Level Pathway (DLP). Regardless of the pathway, all refugees received the aforementioned services (see *Initial Planning and Cooperation*), which were provided by different personnel depending on the service. Such personnel included medical doctors, pediatricians, nursing staff, social workers, psychologists and cultural mediators, the latter being mostly volunteers from the local Ukrainian community of Rome. The main centers were fully operational during the first semester of assistance, working Monday through Sunday from 8 a.m. to 8 p.m., with a decreased intensity of operations after the first semester. On-demand opening was possible for unexpected migrant flows (i.e., refugee buses entering Rome at night).

The CRP consists of three reception hubs, which were set up during the first three weeks of operations and distributed strategically throughout Rome. The first two were located in critical urban confluence points, “Termini Central Station” and “Roma Ostiense Station”, which are among the biggest train stations in Italy. The Termini Hub was set up in collaboration with the Italian Red Cross and the Ostiense Hub with ACEA (Azienda Comunale Energia ed Ambiente, a holding company active in the environmental, energy and water sectors), both through Public Private Partnerships (PPPs). The last center was set up in the Minor Basil of Saint Sophie with the help of the local Christian religious community.

On the other hand, the DLP is composed of small open-access STP ambulatories and Single Access Points spread throughout the six districts that comprise the LHA, where refugees or those helping them were able to request health and social assessments. When needed, the refugees were directed towards the nearest center in the CRP, which also remained available for subsequent visits in case of resurgence of health problems. After the initial assessment, all cases requiring further assistance were forwarded to and analyzed by a central multidisciplinary team of public health professionals, nurses, psychologists and cultural mediators. Based on the specific health need, refugees were directed either to secondary assistance facilities (i.e., pediatric hospital “Ospedale Pediatrico Bambino Gesù”, the national infectious disease hospital “INMI Spallanzani”, outpatient clinics, etc.) to receive treatment or to other support centers appropriate for the specific case, which provided social assistance, food and shelter, and mental health support (i.e., the Health Center for Forced Migrants, Caritas Italiana, etc.). Healthcare information was shared on a case-by-case basis during the referral process to secondary assistance facilities and support centers, with some information being available on different shared databases (i.e., the local vaccination platform).

### Practice challenges and solutions

Many challenges were faced during the implementation phase of the outlined practice guidelines, which required sensible and timely solutions. These challenges include the necessity of rapid resource provision, overcoming linguistic and cultural barriers, guaranteeing a standard of care across multiple sites and coordinating interventions.

To rapidly respond to the oncoming crisis, many resources had to be mobilized to set up the above-mentioned reception centers and to train specific personnel. To this end, many resources were repurposed, especially COVID-19 vaccination hubs (Termini and Ostiense vaccination hubs) and related employees, as welle as medical and Information Technologies (ITs) equipment. Additional resources were acquired through volunteer networks and the local Ukrainian community, which was eager to help. The working relationship between LHA Roma 1 and the local Ukrainian community was mutually beneficial, as it provided the LHA with Ukrainian-speaking cultural mediators, who were hard to find, and allowed the community’s needs to be voiced and better met. The presence of Ukrainian employees helped in smoothing communication issues and solidifying the trusting relationship needed between the Ukrainian cultural minority and the Public Health authorities. This collaboration was also instrumental in the activation of the Minor Basil of Saint Sophie as a reception center, reaching subjects that chose to forego the standardized care pathways and that had contact only with the local Christian religious community.

Given the LHA’s geographical complexity and district heterogeneity, guaranteeing a standard of care across sites was essential. To this end, LHA Roma 1 organized team building activities to form a central multidisciplinary team of Italian and Ukrainian professionals, which included Public Health professionals, nurses, administrative staff and other members, all contributing to the ideation and further development of protocols, documentation and standards of practice. This team was critical in the creation and maintenance of a central data management system and a related database which received data feeds from the daily assistance activities of all hubs and centers, secondary assistance requests for those with specific health conditions and any other concerns relating to daily practice. All data was used according to the European General Data Protection Regulation (GDPR) and Italian law. The database allowed for relevant and timely feedback and resource allocation, resulting in effective multi-site coordination. Furthermore, the team coordinated multiple training sessions, which were provided to social workers and healthcare professionals involved in the delivery of care in the LHA Roma 1 centers dedicated to refugees. Online and on-site training activities were held, concerning the health profile of the Ukrainian population, with specific attention to communicable diseases, immunization status and vaccination attitudes, resulting in greater standardization of practice and better health outcomes.

### Brief overview of assistance data

In the first three months of assistance, starting on the 2nd of March, 9349 refugees were assisted and assigned a STP code, with most of the refugees arriving during the first 5 weeks of operation (74.6%), closely following the forced emigration timeline caused by the conflict [15].

Of all the people assisted, about 70% were females, 40% were minors, and less than 5% were over 65 years of age, which is congruent with the family structures leaving Ukraine. This age and gender distribution further cements the necessity of the partnership with both the local pediatric hospital and volunteering gynecologists in the assistance pathways.

The COVID-19 vaccination status was often undisclosed by refugees, complicating the process of releasing the European COVID-19 certificate “Green Pass’’, which in Italy was required for most public activities until the 1st of May (16). Despite this, more than 2480 COVID-19 vaccine doses were administered to refugees in the reception hubs, mostly to adult females. Furthermore, the 96 (1%) people that were found to be positive with rapid antigen testing were all assisted and isolated in COVID-19 facilities for the appropriate time span.

The tailored care pathway for second level assistance was activated for 206 refugees. The most common reasons were the need for emergency care, life-saving medication (e.g., insulin, transplant medication) and specialty care, which was typically endocrinological (especially hypothyroidism), neurological, cardiovascular (hypertension and heart disease) and pediatric.

## Discussion

The COVID-19 pandemic has shed light on the importance of organizational preparedness and responsiveness to Public Health emergencies, even on a local scale [17–20]. In our context, the already present environment of crisis management facilitated the transition to Ukrainian refugee assistance. A key component of the speed and effectiveness of such a transition was the working relationship with the Ukrainian community. Indeed, it is well known that a cross-cultural interaction ensures better health and social support for minorities (21, 22). Furthermore, the multidisciplinary and multiprofessional connections between medical personnel, nurses, cultural mediators, social workers, psychologists, volunteers, religious organizations and other key actors were essential in ensuring a holistic approach to refugee care. Our experience seems to suggest that future practice should aim to empower local communities and minorities so that their voices are better heard, allowing for more precise and targeted interventions that lead to better long-term public health benefits. In our experience, it was particularly important to consider certain factors: in addition to ensuring access to healthcare (immunizations, diagnostic tests and treatments), multidisciplinary teams made it possible to overcome and mitigate barriers such as language, stigma and the psychological consequences of conflict and exile, access to the internet, the difficulty of having a traceable medical history, which often cause misunderstanding and subsequently low trust in the system [23]. This approach allowed for immediate alignment with the WHO action plan, following the 5 identified priorities: ensuring that migrants and refugees have universal health coverage; the implementation of inclusive health emergency policies; the promotion of social inclusion and reduction of inequalities between people; the strength of migration health governance and data gathering; the support of new partnerships and innovative ways of working [24]. Despite this, the present research in practice article is not without limitations: firstly, our challenges and solutions might be context specific, as different local realities across Italy and Europe might face different pressures and constraints, thus requiring alternative solutions; secondly, even within our Region, LHAs tend to work in siloes, which is why the current practice does not address coordination efforts with other LHAs. Therefore, further studies are required to assess the reproducibility of the approach, its applicability to different contexts and to collaborative efforts between different Local Health Authorities.

Regardless of the limitations, this article provides further insight into organizational efficiency. In this context, sound leadership [25] helps guide the process of modulating and repositioning structures by assigning them different functions, by creating a sense of urgency that motivates trusted individuals to take action. Indeed, as the COVID-19 pandemic has shown, strong leadership is needed to make the best decisions in a short period of time in order to ensure an organization delivers the best care in the best way [26–28]. For example, resource provision is a critical component of an effective emergency response, especially when time is of the essence. National and supranational policies that address this issue often lack specific and practical indications on resource provision, creating an organizational gap that has to be addressed locally. In our context, a sound leadership structure was fundamental for organizational resilience and resource repurposing.

Despite this, Public Health policies cannot entirely rely on organizational excellence, as most nations have heterogeneous landscapes in terms of population needs and resources. The Migrant Integration Policy Index (MIPEX) puts Italy as a “Halfway Favorable” country for what concerns several key indicators of policy integration regarding migrants [29]. Thus, a feedback mechanism should be implemented to create a dynamic relationship between policy and practice [30], to modulate each intervention according to the local environment. This, in turn, would better realize the potential of local realities to provide appropriate health interventions and increase their population’s well-being.

## Conclusion

The necessity of rapid resource provision, overcoming linguistic and cultural barriers, guaranteeing a standard of care across multiple sites and timely coordination of interventions were all challenges faced during LHA Roma 1’s efforts to assist Ukrainian Refugees. Solutions to such challenges involved the formation of Public Private Partnerships, the creation of a centralized multicultural and multidisciplinary team and the collaboration with the local Ukrainian community. A sound central leadership allowed for a unified, coordinated and motivated response within a vision shared amongst all stakeholders. The empowerment of local communities was fundamental to improve the appropriateness of our response and the resilience of our local health ecosystem. This experience highlights the importance of a dynamic relationship between policy and practice, that would allow multiple iteration cycles to improve interventions according to the feedback provided by the local environment, thus fully realizing the potential of each local health ecosystem to provide the best assistance to refugees.

## Data Availability

The data that support the findings of this study are available from ASL Roma 1 (Local Health Authority “Roma 1”) but restrictions apply to the availability of these data, which were used under license for the current study, and so are not publicly available. Data are however available from the authors upon reasonable request and with permission of ASL Roma 1 (Local Health Authority “Roma 1”).

## List of abbreviations

LHA: Local Health Authority
STP: Straniero Temporaneamente Presente - Temporarily Present Foreigner
CRP: Central Reception Pathway
DLP: District Level Pathway
